# Toward improved phosphorus efficiency in monogastrics—interplay of serum, minerals, bone, and immune system after divergent dietary phosphorus supply in swine

**DOI:** 10.1152/ajpregu.00215.2015

**Published:** 2016-03-09

**Authors:** Michael Oster, Franziska Just, Kirsten Büsing, Petra Wolf, Christian Polley, Brigitte Vollmar, Eduard Muráni, Siriluck Ponsuksili, Klaus Wimmers

**Affiliations:** ^1^Leibniz Institute for Farm Animal Biology, Institute for Genome Biology, Dummerstorf, Germany;; ^2^University of Rostock, Nutrition Physiology and Animal Nutrition, Rostock, Germany; and; ^3^University of Rostock, Institute for Experimental Surgery, Rostock, Germany

**Keywords:** bone characteristics, diet, gene expression, peripheral blood mononuclear cell transcriptome, phosphorus

## Abstract

Phosphorus (P) is of vital importance for many aspects of metabolism, including bone mineralization, blood buffering, and energy utilization. In order to identify molecular routes affecting intrinsic P utilization, we address processes covering P intake, uptake, metabolism, and excretion. In particular, the interrelation of bone tissue and immune features is of interest to approximate P intake to animal's physiology and health status. German Landrace piglets received different levels of digestible phosphorus: recommended, higher, or lower amounts. At multiple time points, relevant serum parameters were analyzed and radiologic studies on bone characteristics were performed. Peripheral blood mononuclear cells were collected to assess differential gene expression. Dietary differences were reflected by serum phosphorus, calcium, parathyroid hormone, and vitamin D. Bone reorganization was persistently affected as shown by microstructural parameters, cathepsin K levels, and transcripts associated with bone formation. Moreover, blood expression patterns revealed a link to immune response, highlighting bidirectional loops comprising bone formation and immune features, where the receptor-activator of NF-κB ligand/receptor-activator of NF-κB kinase system may play a prominent role. The modulated P supplementation provoked considerable organismal plasticity. Genes found to be differentially expressed due to variable P supply are involved in pathways relevant to P utilization and are potential candidate genes for improved P efficiency.

in all organisms, phosphorus (P) plays an important role in organic and inorganic compounds and is involved in many aspects of metabolism and growth, including energy utilization, blood buffering, cell membrane bipolarity, kinase-mediated signal transduction, and bone mineralization. In mammals, including pigs, blood P homeostasis is orchestrated by absorption from intestine, bone turnover, and excretion via kidney into urine. These processes are basically mediated by calcitriol [1,25(OH)_2_D_3_], fibroblast growth factor 23 (FGF23), and parathyroid hormone (PTH). Furthermore, bone resorption is controlled by a system comprising soluble RANKL (receptor-activator of NF-κB ligand) and its decoy receptor osteoprotegerin, which is essential for osteoclast differentiation and activity. In turn, bone demineralization is followed by increased serum cathepsin K levels.

Pigs represent valuable experimental models to study the molecular routes influenced by variable supply of minerals and micronutrients (54). Many studies focused on optimized dietary P intake (26, 53, 63), but only a few studies have been published regarding molecular features involved in pathways being responsive for restricted or excessive dietary P levels (12, 31–33). In pigs, P-restricted diets were associated with increased renal mRNA abundances of parathyroid hormone receptor and 1α-hydroxylase, corresponding to decreased PTH and increased circulating calcitriol levels (2). Furthermore, high dietary P levels caused an intracellular redistribution of sodium-dependent inorganic P transporters in kidney cells (43). Moreover, dietary P supply may influence immune features as reviewed elsewhere (29). In this context, calcitriol-mediated responses on different leucocyte subsets were assumed as indicated by shifts between cellular and humoral immune responses (29, 36). Specifically, immune-related genes like *IL-1β*, *NFκB*, and *TEC* appeared to be sensitive for dietary P levels (11, 49, 58).

In addition to these developmental and health aspects, one has to consider also environmental issues. In order to maximize weight gain and optimize bone mineralization, experience has shown that dietary P supply often exceeds age-specific requirements considerably. Consequently, pig manure contains high P load (7, 16), contributing to increased P losses following agricultural drainage and runoff (48, 56, 64), causing serious concerns for both soil and water ecosystems (10, 13, 28, 37, 73). Hence, strategies to minimize the environmental load of pig production have been suggested (1, 69). Thus, efficiency in P turnover becomes important for sustainable food supply, boosted by concerns regarding limited global P resources (20, 51).

Phytic acid is the principal storage form of P in many food plants, cereals, and seeds. Because of the lack of phytases, such P sources can be used by monogastrics only to a small extent. Toward this aim, modern feeding regimens improved dietary P utilization via supplemention of microbial phytase (25, 42, 44), lowering both required dietary P concentration and the pig's fecal P excretion (8, 24). However, further improvements are needed as a result of constantly increased growth performance following contemporary breeding management, disease control, advances in physiology, and selection programs (14, 65). Indeed, genetics potentially has an impact on P utilization in pigs (2, 4, 30).

We aim to identify molecular features and metabolic routes responsive for dietary P intake related to organismal P efficiency and liability to calcium-phosphorus imbalances. Hence, this study investigates effects of diets varying in calcium-phosphorus ratios on growing pigs weighing between 8 and 25 kg. Specifically, the transcriptional response of peripheral blood mononuclear cells (PBMC) was investigated since PBMCs represent physiological and environmentally sensitive transcriptional shifts in other tissues and organs (38, 47). Furthermore, relevant hormone levels and blood parameters were analyzed, and radiologic studies on bone characteristics were performed.

## MATERIALS AND METHODS

### 

#### Animals and diets.

**Fig. 1.**
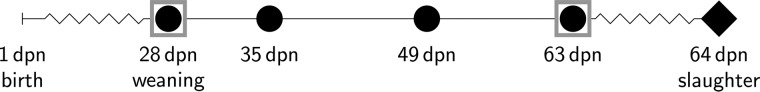
Experimental design. Landrace piglets received low (L), medium (M), or high (H) levels of dietary P postweaning. Blood samples (*n* = 18) were collected at four sampling points, as indicated by the black circles (blood: 28 dpn, 35 dpn, 49 dpn, and 63 dpn). Rectangles indicate time points for transcriptome profiling (28 dpn, 63 dpn). Black diamond indicates femur sampling (64 dpn). dpn, days postnatum

The study was approved by the Scientific Committee of the Leibniz Institute for Farm Animal Biology, and the experimental setup was generally licensed by the Ethics Committee of the federal state of Mecklenburg-Western Pomerania, Germany (Landesamt für Landwirtschaft, Lebensmittelsicherheit und Fischerei; LALLF M-V/TSD/7221.3-2.1-020/09). The experimental design is shown in Figure 1. In total, eighteen German Landrace piglets obtained from three litters were randomly assigned to one of three wheat/barley/soybean-based diets varying in digestible P (see Supplemental Table S1). Hence, the diets differed in their calcium-phosphorus ratios. From weaning [28 days postnatum (dpn)] until slaughter (64 dpn), piglets received a diet containing low P (L), medium P (M), or high P (H) levels. Neither phytase nor other phosphatases were added. The achieved levels of soluble P were 0.33% (L), 0.51% (M), and 0.74% (H), respectively. Piglets were individually reared in cages on flat decks in environmentally controlled rooms. The animals had ad libitum access to pelleted feed and water.

#### Collection and preparation of serum samples.

Blood samples were collected from Vena cava cranialis into heparin-coated tubes at four time points and stored on ice (28 dpn, 35 dpn, 49 dpn, 63 dpn). Serum was prepared and samples were stored at −80°C until use.

#### Physiological parameters, hormones, and blood cell parameters.

Serum samples obtained from animals at 28 dpn, 35 dpn, 49 dpn, and 63 dpn were used. Physiological parameters (inorganic phosphorus, calcium, alkaline phosphatase, magnesium, creatinine, albumin, and glucose) were analyzed with commercial assays using Fuji DriChem 4000i (FujiFilm, Minato, Japan). Serum parathyroid hormone (Immundiagnostik, Bensheim, Germany), calcidiol (IBL, Hamburg, Germany), FGF23 (BlueGene Biotech, Shanghai, China), osteocalcin (Cloud-Clone, Houston, TX), soluble RANKL (Biomedica, Vienna, Austria), and cathepsin K (Biomedica) were determined in duplicate using commercially available ELISA, according to the manufacturer's protocols. Calcitriol was determined via chemiluminescent immunoassay, according to the manufacturer's protocols (DiaSorin, Saluggia, Italy). The intra-assay and interassay coefficients of variation were calculated, i.e., parathyroid hormone (5.6%; 5.9%), calcidiol (6.0%; 3.0%), sRANKL (6.2%; 18.5%), cathepsin K (6.8%; 7.2%), osteocalcin (9.2%; 13.0%), and FGF23 (9.1%; 6.2%), respectively. The intra-assay coefficient of variation (%) in calcitriol was 1.7. Additionally, blood cell counts (leucocytes, erythrocytes, and platelets) were analyzed (ABX Pentra 60, HORIBA ABX SAS, Montpellier, France). Mean values of above-mentioned measurements are given in Supplemental Table S2.

#### Micro-CT measurement of bone characteristics.

**Fig. 2.**
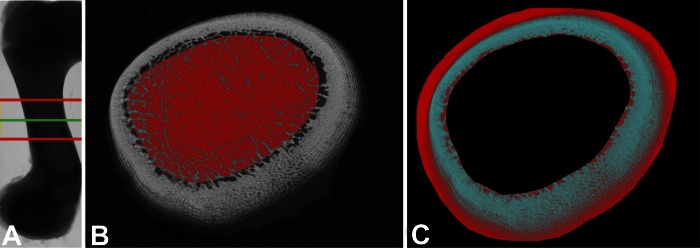
Representative images of selected volume of interest (VOI) in femur (64 dpn). *A*: green line marks the reference line representing the middle region of the bone. Red lines mark upper and lower border, respectively. Red area marks selected region of interest (ROI), representing trabecular (*B*) and cortical (*C*) bone, respectively.

The left femur of each piglet was sampled at 64 dpn and stored in Formalin (4°C) until use. Radiologic studies were performed using a high-resolution micro-CT Imaging System (SkyScan 1076, Bruker-MICROCT, Kontich, Belgium). Before being scanned, samples were cleaned in water and stored in 0.9% saline solution at 4°C for at least 24 h. To prevent dehydration during the scanning process, bone samples were wrapped in cling film. For all measurements, the following criteria were used: X-ray source voltage/amperage: 70 kV/141 μA, filter: aluminium 1.0 mm, pixel size: 18 μm, pixel matrix: 4,000 × 2,672, rotation step: 0.6°, averaging frame: 4. After the scanning process, the digital raw images were reconstructed by using NRecon (SkyScan, Kontich, Belgium), a specialized software using cone beam volumetric algorithms. Regarding the middle region of the bone, the volume of interest (VOI) covered each 500 slices in proximal and distal direction, respectively (Fig. 2*A*). The following parameters were used to reconstruct each sample: defect pixel masking: 20%; beam-hardening: 30%, individual misalignment compensation, and fixed histogram thresholds. Subsequently, a trabecular polygonal region of interest (ROI) representing the bone marrow (Fig. 2*B*) and a cortical bagel-like ROI across the whole VOI (Fig. 2*C*) were defined by using CT-analyzer. Artificial phantom rods, containing 0.25 g and 0.75 g calcium-hydroxylapatite·cm^−3^, respectively, were used for calibration. Trabecular bone mineral density (BMD) and tissue mineral density (TMD) were measured. In addition, a three-dimensional analysis was performed to obtain microstructural parameters, including trabecular bone volume/tissue volume ratio (BV/TV), trabecular number (TbN), trabecular separation (TbSp), trabecular thickness (TbTh), and structure model index (SMI).

#### RNA isolation, target preparation, and hybridization.

Samples obtained at 28 dpn and 63 dpn were used to isolate peripheral blood mononuclear cells (PBMC) from 5 ml of blood by centrifugation on a Histopaque density gradient (Sigma-Aldrich, Taufkirchen, Germany), and then stored at −80°C. Total RNA was isolated using Qiazol reagent per the manufacturer's directions (Qiagen, Hilden, Germany). Quantification and purification were performed as previously described (52). All RNA samples were stored at −80°C until transcriptome profiling was performed. Individual RNA samples were transcribed to DNA using the Ambion WT expression kit (Ambion, Austin, TX). DNA preparations were fragmented and labeled using the WT terminal labeling kit (Affymetrix, Santa Clara, CA). Subsequently, preparations were hybridized on genome-wide “snowball” arrays (Affymetrix, Santa Clara, CA), a platform invented for genome-wide analyses of the pig transcriptome (23). Raw data were generated using Affymetrix GCOS 1.1.1 software and were deposited in a MIAME-compliant database (19), the National Center for Biotechnology Information Gene Expression Omnibus (www.ncbi.nlm.nih.gov/geo; accession number: GSE66308).

#### Data analyses.

Arrays passed the appropriate quality control criteria, as proposed previously (35). The data were RMA normalized (Log2) and filtered by both standard deviation (SD > 0.23) and mean (*m* > 2.5). All samples taken at 28 dpn were considered to represent the physiological untreated state and were, therefore, assigned as reference samples (R). Hence, transcriptional effects were analyzed between samples obtained from animals fed L, M, and H diets at 63 dpn and the reference. Relative changes of mRNA abundances were estimated via variance analyses (SAS Institute, Cary, NC), including effects represented by dietary group, sire, and sex [*V*_ijk_ = μ + dietary group_i_ + sire_j_ + sex_k_ + error_ijk_]. To control for multiple testing, *P* values were converted to a set of *q* values (68). The level of significance was set at *P* ≤ 0.05 and *q* ≤ 0.05, respectively. Annotation data for Affymetrix snowball arrays were obtained from the developers (23). Lists of altered transcripts were evaluated with Ingenuity Pathway Analysis (IPA; Ingenuity Systems, Redwood City, CA). The significance of association between data set and pathway was set at *P* ≤ 0.05.

Changes for bone characteristics, physiological parameters, and hormones were estimated via variance analyses (SAS Institute), including effects represented by dietary group, sire, and sex, as done for transcriptomic data. Differences were considered significant at *P* ≤ 0.05.

## RESULTS

The study investigates effects of wheat/barley-based diets varying in calcium-phosphorus ratios on growing pigs. Clues for metabolic alterations were deduced from blood metabolites, bone characteristics, and transcriptome profiles of PBMC. With regard to environmental and economic issues, the objectives of this study were to identify P-responsive metabolic routes and molecular features in growing pigs.

### 

#### Hormone levels and blood metabolites.

**Fig. 3.**
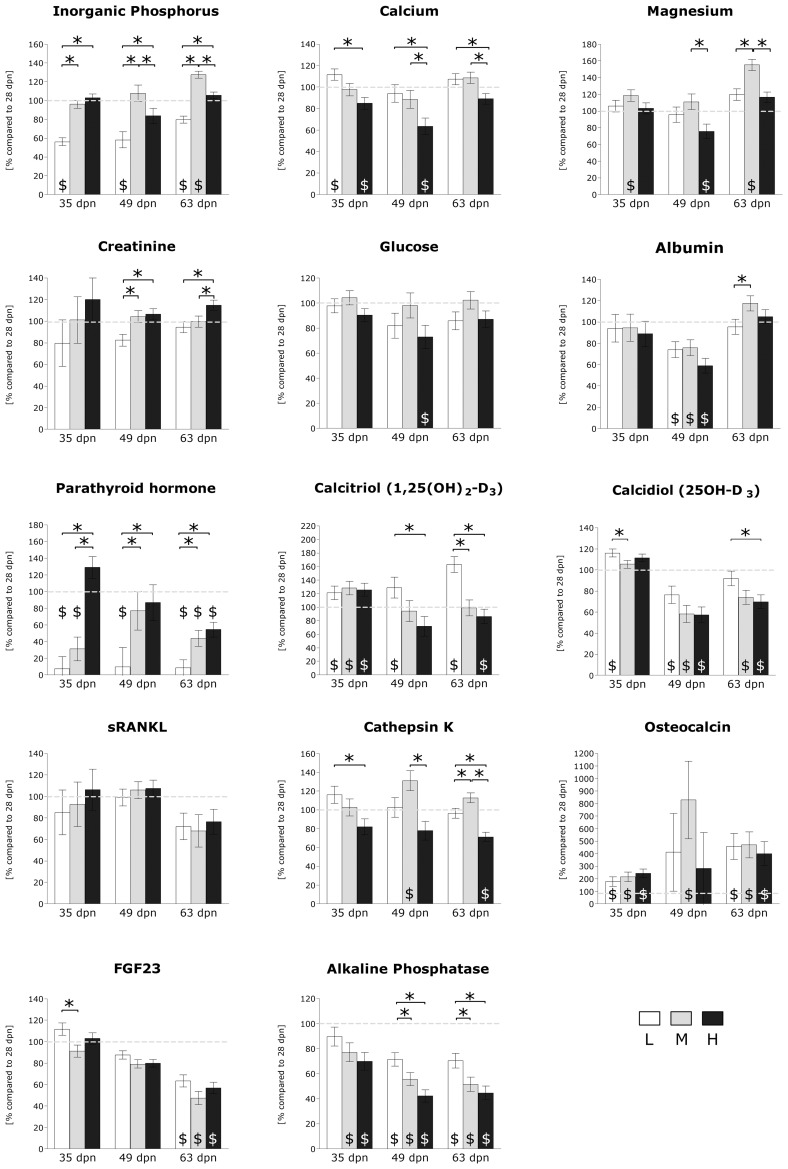
Hormone levels and blood metabolites. Relative levels (least square means ± SE) of serum inorganic phosphorus, calcium, magnesium, creatinine, glucose, albumin, parathyroid hormone, calcitriol, calcidiol, sRANKL, cathepsin K, osteocalcin, FGF23, and alkaline phosphatase were shown in comparison to untreated samples (28 dpn). L: white bars. M: gray bars. H: black bars. Dietary effects within stage: *P ≤ 0.05. Stage effect within dietary group: $*P* ≤ 0.05. dpn, days postnatum.

**Fig. 4.**
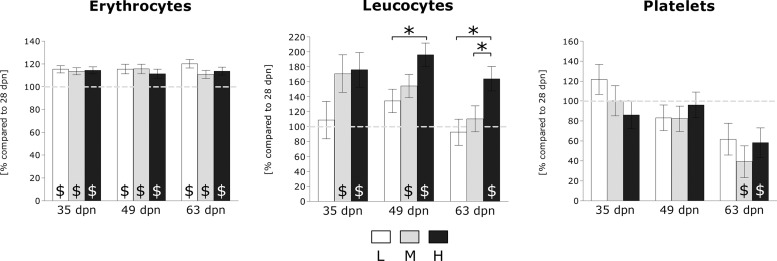
Blood cell counts. Relative levels (least square means ± SE) of erythrocytes, leucocytes, and platelets were shown in comparison to untreated samples (28 dpn). L: white bars. M: gray bars. H: black bars. Dietary effects within stage: *P ≤ 0.05. Stage effect within dietary group: $*P* ≤ 0.05. dpn, days postnatum

Physiological serum parameters were altered as a result of both stage and dietary treatment (Fig. 3). Levels of inorganic phosphorus differed between L and M samples at 35 dpn, 49 dpn, and 63 dpn (M > L). Furthermore, H samples showed higher levels than L samples at 35 dpn, 49 dpn, and 63 dpn (H > L). At 63 dpn, inorganic P levels were found to be increased in M compared with H samples (M > H). Calcium levels differed between L and H samples at 35 dpn, 49 dpn, and 63 dpn (L > H). Furthermore, M samples showed higher levels than H samples at 49 dpn, and 63 dpn (M > H). Magnesium levels were found to be increased in M samples at 49 dpn (M > H) and 63 dpn (M > L; M > H). Creatinine levels differed at 49 dpn (H > L; M > L) and 63 dpn (H > L; H > M). Glucose and albumin levels remained unchanged by diets. Regarding PTH, nutrient-related effects within stages were observed between L and H samples at 35 dpn, 49 dpn, and 63 dpn (H > L). In addition, L samples were found to exhibit lower PTH levels than M samples at stages 49 dpn and 63 dpn (M > L), respectively. Moreover, H samples showed increased PTH levels compared with M samples at stage 35 dpn (H > M). Regarding calcitriol, increased levels were found in L samples at 49 dpn (L > H) and 63 dpn (L > H; L > M). Additionally, serum calcidiol levels differed at 35 dpn (L > M) and 63 dpn (L > H). FGF23 levels were increased in L samples at 35 dpn. Osteocalcin and soluble RANKL levels were found to be unaffected by dietary P intake. Cathepsin K levels differed at 35 dpn (L > H), 48 dpn (M > H), and 63 dpn (L > H; M > H; M > L). Regarding alkaline phosphatase, diet-dependent effects within stages were observed between L and M samples (L > M), as well as between L and H samples (L > H) at 49 dpn and 63 dpn, respectively. Relative leucocyte number was found to be increased in H samples at 49 dpn (H > L) and 63 dpn (H > L; H > M) (Fig. 4). No dietary effect on erythrocytes and platelets were observed.

#### Bone characteristics.

At 64 dpn, BMD and SMI were increased in H samples compared with L samples (H > L; Table 1). Furthermore, BV/TV was increased in H samples (H > L; H > M). In L samples, ThN was lowered and the ThSp was increased, when compared with H and M samples, respectively. No dietary differences were observed regarding TMD and TbTh, respectively.

**Table 1.** Bone characteristics of pigs fed experimental diets with low (0.33%), adequate (0.51%), and high (0.74%) digestible P content

Item	Unit	Low		Medium		High	
		LS Mean	SE	LS Mean	SE	LS Mean	SE
Tissue mineral density	g/mm^3^	1.09	0.01	1.09	0.01	1.11	0.01
Bone mineral density	g/mm^3^	0.15^a^	0.01	0.18^a^	0.01	0.21^b^	0.01
Structure model index		0.77^a^	0.22	1.37^a,b^	0.22	1.63^b^	0.21
Bone volume/Total volume	%	17.82^a^	1.31	20.02^a,b^	1.31	21.96^b^	1.23
Trabecular number	1/mm	0.56^a^	0.06	0.70^a,b^	0.06	0.72^b^	0.05
Trabecular separation	mm	3.22^b^	0.33	1.78^a^	0.33	2.05^a^	0.31
Trabecular thickness	mm	0.32	0.02	0.29	0.02	0.32	0.02

a,b,c Letters that differ indicate significant differences between groups (*P* ≤ 0.05).

#### Transcriptome profiles in PBMC.

The snowball microarray covers 47,845 probe sets corresponding to 17,964 annotated genes. After filtering as described above, 27,596 probe sets (∼58 %) remained for analysis. These probe sets corresponded to 15,488 annotated genes.

Transcriptional differences were pronounced due to effects mediated by both dietary challenge and age. Hence, the analyses focused on transcripts representing longitudinal transcriptional patterns specific for L, M, or H samples, including the physiologically untreated samples as reference samples (R). Between L and R samples, seven probe sets (6 probe sets L > R) were found to be altered (Supplemental Table S3). Comparing M and R samples, four probe sets (three probe sets M > R) showed an altered abundance. Between H and R samples, 687 probe-sets (563 probe sets H > R) differed significantly. Of these, 678 probe sets (555 probe sets H > R) were private to the comparison between H and R and were used for further analysis (Table 2). Specifically, pathways associated with bone metabolism exhibited diet-specific expression, including RANK signaling and BMP signaling. Furthermore, a set of immune pathways was found to differ between H and R samples, in particular, transcripts associated with NF-κB signaling, glucocorticoid receptor signaling, p38 MAPK signaling, regulation of the epithelial-mesenchymal transition pathway, TREM1 signaling, FGF signaling, NGF signaling, B cell receptor signaling, and TGF-β signaling. Moreover, IPA proposed that the altered RANK signaling rather contributes to cell survival than osteoclast differentiation. Moreover, a stimulation of T-cell proliferation was predicted in H samples (*z*-score = 2.483; 51 genes; overlap *P* = 3.13·E^−11^).

**Table 2.** Altered pathways between H and R samples in PBMC

Regulated Pathway	No. of genes	Involved Genes
B-cell receptor signaling‡	13	BCL6 (+1.79); CREB5 (+1.66); CREBBP (+1.28); GSK3B (+1.30); MAP3K2 (+1.77); MAP3K3 (+1.40); MAP3K5 (+1.44); MAPK1 (+1.30); MAPK14 (+1.49); NFAT5 (+1.36); PIK3CG (+1.37); SOS2 (+1.65); SYK (+1.68)
BMP signaling*	6	BMP8A (−1.26); CREBBP (+1.28); MAPK1 (+1.30); MAPK14 (+1.49); RUNX2 (+1.29); SMAD5 (+1.37)
FGF signaling‡	9	CREB5 (+1.66); CREBBP (+1.28); CRKL (+1.29); HGF (+1.88); MAP3K5 (+1.44); MAPK1 (+1.30); MAPK14 (+1.49); PIK3CG (+1.37); SOS2 (+1.65)
Glucocorticoid receptor signaling*	19	A2M (+1.71); ANXA1 (+1.54); CREBBP (+1.28); DUSP1 (+1.77); FKBP5 (+1.49); GTF2A1 (+1.37); GTF2A2 (+1.30); IL1B (+1.47); IL1R2 (+2.49); MAPK1 (+1.30); MAPK14 (+1.49); NCOA2 (+1.35); NFAT5 (+1.36); PIK3CG (+1.37); SGK1 (+1.98); SOS2 (+1.65); STAT5B (+1.38); TGFBR1 (+1.42); TSC22D3 (+1.41)
NF-κB signaling‡	15	CREBBP (+1.28); GSK3B (+1.30); IGF2R (+1.33); IL1B (+1.47); IL1R1 (+1.73); IL1R2 (+2.49); IRAK4 (+1.28); MAP3K3 (+1.40); PIK3CG (+1.37); RANK (+1.34); TAB3 (+1.36); TANK (+1.52); TGFBR1 (+1.42); TLR2 (+1.61); TLR4 (+1.88)
NGF signaling‡	10	CREB5 (+1.66); CREBBP (+1.28); MAP3K2 (+1.77); MAP3K3 (+1.40); MAP3K5 (+1.44); MAPK1 (+1.30); PIK3CG (+1.37); ROCK1 (+1.48); RPS6KA3; (+1.50) SOS2 (+1.65)
p38 MAPK signaling‡	12	CREB5 (+1.66); CREBBP (+1.28); DUSP1 (+1.77); IL1B (+1.47); IL1R1 (+1.73); IL1R2 (+2.49); IRAK4 (+1.28); MAP3K5 (+1.44); MAPK14 (+1.49); MEF2A (+1.30); RPS6KA3 (+1.50); TGFBR1 (+1.42)
RANK signaling†	7	MAP3K2 (+1.77); MAP3K3 (+1.40); MAP3K5 (+1.44); MAPK1 (+1.30); MAPK14 (+1.49); PIK3CG (+1.37); RANK (+1.34)
Regulation of the epithelial-mesenchymal transition pathway‡	15	APH1B (+1.44); FZD4 (+2.08); GSK3B (+1.30); HGF (+1.88); ID2 (+1.43); MAPK1 (+1.30); MMP2 (+1.41); PIK3CG (+1.37); PSEN1 (+1.38); PYGO1 (+2.09); RBPJ (+1.27); SOS2 (+1.65); TGFBR1 (+1.42); WNT1 (−1.36); ZEB2 (+1.47)
TREM1 signaling‡	9	CD86 (+1.62); IL1B (+1.47); LAT2 (+1.47); MAPK1 (+1.30); NLRP3 (+1.52); STAT5B (+1.38); TLR2 (+1.61); TLR4 (+1.88); TYROBP (+1.71)

Fold changes in parentheses indicate positive (H>R) or negative (H<R) transcript abundance. Enrichment is significantly different from those of R samples:

* *P* ≤ 0.05,

† *P* ≤ 0.01,

‡ *P* ≤ 0.001.

## DISCUSSION

Mature and aging organisms exhibit ongoing in- and e-fluxes of P largely buffered by the osseous storage. Adolescent pigs are characterized by a high daily weight gain depending on sufficient P supply and favoring P accumulation. Maintenance and gain require a number of well-matched regulating processes for maintaining metabolic homeostasis (55), including various changes in gene expression and hormone levels. Increased efficiency of P utilization is desirable because of resource saving and environmental issues. Modulating the P supply in an experimental model points to those molecular routes that are responsive to dietary challenges representing pathways, molecules, and genes related to P efficiency. Therefore, the experiment contributes toward the implementation of strategies to preserve global resources and to reduce environmental load to soil and water ecosystems.

### 

#### Hormone and mineral response to variations in dietary P supply.

The experimental groups exhibited considerable diet-related changes of serum mineral and hormone levels, indicating that the dietary variations in P supply caused shifts of major players of P homeostasis. In particular, inorganic P levels were persistently lowered in L samples, whereas calcium levels were persistently lowered in H samples. These observations are consistent with previous studies investigating mineral homoeostasis (2, 3, 41). As reviewed elsewhere, calcium and P serum levels are counterbalanced between enteral absorption, osseous mobilization, and renal excretion rates (9), which are coordinated by several endocrine factors, including PTH and calcitriol (18, 71). Consistent with previous studies (21, 60, 67, 76), lowered PTH levels, but increased vitamin D levels, reflect the organismal effort to minimize urinary P losses and to enhance enteral P absorption in L samples. Specifically, the intestine was highlighted as an initial site maintaining mineral homoeostasis via enhancing a sodium-coupled P cotransport system (22, 27, 43, 45, 61). Additionally, bone-derived FGF23 acts on blood P homeostasis. The short-term increase of FGF23 might account to feedback loops interrelating absorption, utilization, and excretion, as reviewed elsewhere (59). However, the absence of long-term differences is rather surprising since affected FGF23 levels have been observed in humans and rodents due to diets varying in P (59). The different observations between studies may be due to species-specific endocrine characteristics, or, more likely, may represent differences of FGF23 ELISAs with variable or unknown specificity for intact FGF23 (iFGF23), COOH-terminal FGF23 (cFGF23), or total FGF23 (tFGF23; i.e., iFGF23 and cFGF23). Regarding the porcine ELISA used here, there is no information provided about whether cFGF23, iFGF23, or tFGF23 is detected; the measured concentrations, however, indicate that tFGF23 was detected. ELISA-based measures of iFGF23 or cFGF23 show different degrees of intraindividual and interindividual variation, highlighting cFGF23 as a more reliable diagnostic biomarker for P homeostasis and renal impairment (66). In addition to superior endocrine responses, increased abundances of serum alkaline phosphatase may contribute to mobilize P resources in L samples. In contrast, H samples obviously aimed to maximize renal P losses by a strong increase of PTH at 35 dpn, indicating that kidneys are involved in physiological short-term responses. Urine sampling was not possible due to stable facilities and appropriate farm animal husbandry to verify dietary phosphate absorption on different diets. However, changes of calcitriol and expression of renal Cyp27a1 were consistent with phosphate deprivation on the L diet (data not shown).

Regarding the hormonal orchestration of mineral turnover, the balance of osteoblast- and osteoclast-mediated actions are of interest. Moreover, bone is involved in determination, generation, and maturation of bone marrow-derived immune cell, during which signals linking immune cells and osteocytes are evident (70). In fact, *RUNX2*, a key regulator of osteoblast differentiation in mammalian cells (46, 77), showed increased mRNA abundances in H samples. Since physiological levels of *RUNX2* induce expression of principal osteoblast-specific genes (17, 40), its increased mRNA abundance may be positively involved in bone formation. Osteocalcin abundances were unaffected by diet, suggesting comparable osteoblast activity (57, 74). Furthermore, no dietary effects were observed regarding sRANKL, which is known to bind to preosteoclasts and induce osteoclast differentiation. However, H samples showed reduced serum levels of cathepsin K, the major collagenous protease involved in bone-resorption secreted by mature osteoclasts (62). Hence, H animals might be characterized by a lowered bone resorption rate. Obviously, the balance of bone formation (osteoblast-mediated) and bone resorption (osteoclast-mediated) was altered by dietary P supply, suggesting an increased mineralization in H samples. Indeed, this issue was mirrored in several bone characteristics, including increased values for BMD, BV/TV, SMI, and TbN. Consequently, the TbSp was lowered. In order to induce a persistent negative P balance, H samples might favor an increased trabecular bone growth and osteocalcin-independent effects on bone mineralization. However, the mineralization effects seem to affect the trabecular bone and its microstructural morphology only. To what extent increased trabecular bone impacts bony strength needs to be evaluated through further biomechanical testing. It should be noted that levels of albumin and glucose remained unaffected by diets, indicating regular nutrient utilization. However, different time-dependent alterations of serum creatinine may account for renal perturbations in L and H samples, respectively.

#### Transcriptional responses due to high P supply.

The PBMC expression patterns revealed considerable shifts due to high P supply. The mRNA abundances of RANK signaling and BMP signaling were increased, highlighting implications in osteoblast/osteoclast cross talk in order to maintain mineral homoeostasis. Thus, gene expression in PBMCs reflected the observed diet-dependent structural responses of osseous tissue in H samples. Furthermore, H samples exhibited a number of primarily inflammatory pathways, which are partly depicted previously to be P-responsive (11). In fact, the P-specific expression pattern suggests implications beyond osseal alterations regarding an impact on immune status and health due to the strong increase of transcripts related to pattern recognition. In this context, bone marrow niches as a specialized microenvironment modulating both bone formation and immune features, according to organismal requirements and external stimuli, are of interest. Indeed, bone remodeling has been proven to have an impact on hematopoiesis and immune features (6, 39).

Furthermore, RANKL and its signal receptor RANK are essential for osteoclastogenesis (50). Moreover, mice lacking functional *RANK* exhibited perturbed immune features, including splenic B-cell deficiency and lack of peripheral lymph nodes (15, 34). In our study, the unaltered serum RANKL level supports the result obtained from IPA proposing that the transcriptional alterations in RANK signaling do not necessarily impact on osteoclast differentiation but rather contribute to cell survival, an issue, which may act on immune competent cells expressing *RANK*, e.g., dendritic cells (75). Indeed, the diet-specific expression pattern reflects an enrichment of dendritic cells and corresponding precursor cells in H animals (see Supplemental Table S3). Because the RANKL/RANK system significantly contributes to regulate the interaction between dendritic cells and CD4+ T cells expressing *RANKL* (72), the observed expression patterns might stimulate T-cell proliferation in H animals (5). Interestingly, our PBMC expression analyses revealed that the proliferation of T lymphocytes was predicted to be increased in H samples. Indeed, dietary P supply seems to stimulate T lymphocytes, as previously shown via mitogen-induced lymphocyte proliferation assays (36). Incidentally, activated T cells are capable of potently promoting and suppressing the reorganization of osseous tissue (70, 75). Hence, the study suggests possible RANKL/RANK-mediated effects on the bidirectional crosstalk between bone and immune features due to high P supply. Although it is not yet possible to assign distinct molecular routes of causality, these results highlight RANK as a gatekeeper in P utilization, orchestrating P homoeostasis and immune features.

### Perspectives and Significance

Diet-specific serum hormone and mineral alterations display responses regarding variations in P supply, which indicate a regulatory involvement of major factors contributing to the maintenance of P homeostasis. The endocrine system revealed diet-specific organismal efforts to minimize P losses (L samples) or to induce a negative P balance (H samples), respectively. Therefore, the dietary challenge revealed considerable developmental plasticity in pigs. Diet-dependent endocrine modulations go with PBMC expression patterns as observed in H samples, where genes involved in pathways relevant to P utilization partly reflected the reorganization of osseous tissue. Additionally, the transcriptional shifts of pathways related to acquired and innate immunity implies a trade-off between micronutrient supply and immune system. The data suggest that RANK might contribute to link P homoeostasis and immune features, thereby contributing to the reorganization of osseous tissue and orchestrating the expression of immunity-related transcription factors. These issues underscore the need to redefine animal's P requirement in order to make full use of breeding highly efficient livestock.

## GRANTS

This work was partly funded by the Leibniz Science Campus Phosphorus Research Rostock and the FP7 EU-project ECO-FCE. The Leibniz Institute for Farm Animal Biology provided its own matched funding. The funders had no role in study design, data collection and analysis, decision to publish, or preparation of the manuscript.

## DISCLOSURES

No conflicts of interest, financial or otherwise, are declared by the authors.

## AUTHOR CONTRIBUTIONS

Author contributions: M.O., K.B., B.V., and K.W. conception and design of research; M.O., F.J., K.B., C.P., and K.W. performed experiments; M.O., F.J., K.B., P.W., C.P., B.V., E.M., S.P., and K.W. analyzed data; M.O., K.B., P.W., B.V., and K.W. interpreted results of experiments; M.O. and K.W. drafted manuscript; M.O., F.J., K.B., P.W., C.P., B.V., E.M., S.P., and K.W. edited and revised manuscript; M.O., F.J., K.B., P.W., C.P., B.V., E.M., S.P., and K.W. approved final version of manuscript; M.O. and C.P. prepared figures.

## Supplementary Material

Supporting Information Table S2

Supporting Information Table S3

Supporting Information Table S1
